# Disitamab vedotin (RC48) combined with PD-1 inhibitors in locally advanced or metastatic urothelial carcinoma: clinical outcomes and prognostic factors from a multicenter real-world study

**DOI:** 10.3389/fimmu.2026.1815591

**Published:** 2026-04-29

**Authors:** Xin Chang Zou, Lie Yu Xu, Yu Yang Yuan, Gu Yue Zhang, Jian Biao Huang, Tao Zeng

**Affiliations:** 1 Department of Urology, Second Affiliated Hospital of Nanchang University, Nanchang, China; 2 Department of Urology, Jiangxi Provincial People’s Hospital, Nanchang, China; 3 Department of Urology, First Affiliated Hospital of Nanchang University, Nanchang, China; 4 Department of Urology, First People’s Hospital of Jiujiang City, Jiujiang, China; 5 Jiangxi Cancer Hospital and Institute, Jiangxi Clinical Research Center for Cancer, The Second Affiliated Hospital of Nanchang Medical College, Nanchang, China

**Keywords:** disitamab vedotin, locally advanced or metastatic urothelial carcinoma, mixed patient population, PD-1 inhibitor, real-world study

## Abstract

**Background and objectives:**

The combination of disitamab vedotin (RC48) with PD-1 inhibitors has shown synergistic potential in preclinical studies for treating advanced urothelial carcinoma (UC). Nevertheless, real-world evidence regarding its clinical efficacy and safety profile remains limited. This multicenter real-world study aims to comprehensively evaluate the therapeutic outcomes and safety of RC48 combined with PD-1 inhibitors in patients with locally advanced or metastatic urothelial carcinoma (La/mUC), with a particular focus on analyzing the impact of comorbidities on treatment efficacy and prognosis.

**Patients and methods:**

A retrospective analysis was conducted on 132 patients with La/mUC who received RC48 combined with a PD-1 inhibitor at six treatment centers between June 2022 and March 2024. Clinical–pathological characteristics, treatment regimens, and follow-up data were collected from electronic medical record systems. Efficacy outcomes included progression-free survival (PFS), overall survival (OS), objective response rate (ORR), and disease control rate (DCR). Safety was assessed by recording treatment-related adverse events (TRAEs), with efficacy evaluated across different disease subpopulations. Survival curves were generated using the Kaplan–Meier method, and Cox proportional hazards regression was employed for survival analysis.

**Results:**

A total of 132 patients with La/mUC were enrolled. The RC48 plus PD-1 inhibitor regimen demonstrated an ORR of 71.21% (95% CI: 62.97%–78.25%) and a DCR of 88.64% (95% CI: 82.10%–92.99%). The median PFS was 21 months (95% CI: 14–24 months), and the median OS was not reached. Kaplan–Meier analyses indicated that comorbid conditions such as hypertension, diabetes, hyperlipidemia, or renal insufficiency did not compromise efficacy, with similar PFS and OS observed across subgroups. The most common TRAEs were fatigue (38.64%), nausea (35.61%), anemia (32.58%), pruritus (28.79%), and peripheral neuropathy (23.48%). The incidence of grade 3 adverse events was 13.64%, with no grade 4 or higher events reported.

**Conclusions:**

The RC48 plus PD-1 inhibitor treatment regimen demonstrated clear antitumor activity and manageable safety in a multicenter real-world study of patients with La/mUC, with consistent therapeutic effects observed in patients with comorbidities.

## Introduction

Urothelial carcinoma is a common malignant tumor of the urinary system, and bladder cancer accounts for approximately 90%–95% of cases. Globally, there are approximately 614,000 new bladder cancer cases and 221,000 deaths annually (1, 2). This disease is highly malignant, particularly in patients with locally advanced or metastatic urothelial carcinoma, who have an extremely poor prognosis. The 5-year overall survival rate remains low, and the 5-year survival rate for metastatic urothelial carcinoma has fallen below 5% (3–5). Over the past four decades, platinum-based combination chemotherapy has been the first-line standard treatment for advanced urothelial carcinoma. However, approximately 50% of patients cannot tolerate it due to severe adverse effects such as renal impairment and hearing loss or poor performance status. Moreover, its efficacy is limited in terms of response rate, duration, and survival benefit (6, 7). Therefore, exploring more effective and better-tolerated treatment strategies has become an urgent clinical need in this field.

In recent years, breakthrough progress has been made in precision therapies, exemplified by immune checkpoint inhibitors (ICIs) and antibody–drug conjugates (ADCs), transforming the treatment landscape for advanced urothelial carcinoma (8, 9). ICIs restore T-cell antitumor activity by blocking immune checkpoint pathways such as PD-1/PD-L1 and have become an important treatment option for advanced urothelial carcinoma (10). ADCs precisely target tumor cells through antibodies and release highly potent cytotoxic payloads intracellularly, achieving effective treatment with low toxicity (11). Studies have shown that the payloads of ADCs (such as MMAE) can not only directly kill tumor cells but also induce immunogenic cell death, thereby exhibiting synergistic potential with ICIs (12).

Key clinical studies have provided robust evidence for this combination strategy. The EV-302 study confirmed the efficacy of enfortumab vedotin (a nectin-4-targeting ADC) in combination with pembrolizumab (a PD-1 inhibitor), establishing it as a new standard first-line regimen (13). Likewise, for the domestically developed HER2-targeted ADC drug RC48 (disitamab vedotin), the Ib/II phase study demonstrated that its combination with toripalimab achieved an objective response rate of 73.2% and a median progression-free survival of 9.3 months in treating advanced urothelial carcinoma patients who were either treatment-naive or chemotherapy-refractory, showcasing remarkable antitumor activity (14).

Although pivotal clinical trials have confirmed its efficacy, the stringent inclusion and exclusion criteria may limit the generalizability of the results to the more heterogeneous real-world patient population in clinical practice. Therefore, we conducted this multicenter real-world study to validate the actual efficacy and safety of the RC48 plus PD-1 inhibitor regimen in real-world patients.

Ge H et al. recently demonstrated the efficacy of RC48 in combination with PD-1 inhibitors for advanced urothelial carcinoma in a real-world study (30). In contrast to the study by Ge H et al., which focused on overall efficacy, this study specifically evaluates whether prevalent comorbidities affect therapeutic outcomes and prognosis in patients treated with RC48 plus PD-1 inhibitors. The findings have practical implications for clinical decision-making in real-world, complex patient populations, underscoring the study’s relevance to practice.

## Patients and methods

### Study design

This study is a multicenter, retrospective, real-world investigation conducted across six institutions, including the First Affiliated Hospital of Nanchang University, the Second Affiliated Hospital of Nanchang University, Jiangxi Provincial People’s Hospital, Jiangxi Cancer Hospital, the First Affiliated Hospital of Gannan Medical University, and the Jiujiang First People’s Hospital. The study protocol was approved by the ethics committee of each participating center and was conducted in accordance with the Declaration of Helsinki. Due to the retrospective nature of the study, the requirement for informed consent was waived.

### Patients

Patients with locally advanced or metastatic urothelial carcinoma (La/mUC) who received RC48 combined with a PD-1 inhibitor at the participating centers between June 2022 and March 2024 were retrospectively enrolled in this study. The inclusion criteria were as follows: 1) age 18 years or older, 2) histologically confirmed diagnosis of urothelial carcinoma, 3) disease stage classified as locally advanced (T3–T4) with regional lymph node involvement or distant metastasis (M1), and 4) completion of at least two cycles of the combined treatment regimen. The exclusion criteria included 1) insufficient treatment cycles, 2) absence of critical clinicopathological data, 3) presence of other concurrent active malignancies, and 4) incomplete follow-up imaging data or loss to follow-up precluding outcome assessment.

### Treatment plan and data collection

All patients received RC48 in combination with PD-1 inhibitors. The specific regimen was as follows: RC48 was administered intravenously at 2.0 mg/kg every 2 weeks; PD-1 inhibitors were given every 3 weeks, with toripalimab at 240 mg and tislelizumab at 200 mg. RC48 was typically administered on the first day, followed by sequential infusion of PD-1 inhibitors on the second or third day. Treatment continued until disease progression, intolerable toxicity, patient death, or other reasons for discontinuation.

Follow-up was conducted through medical record review, outpatient visits, and telephone interviews. After treatment completion, patients were followed every 6–8 weeks (data cutoff date: 30 June 2025). Imaging evaluations were performed at baseline and every 2–3 treatment cycles, including CT scans of primary and metastatic lesions, bone scans, and MRI. Imaging data were initially interpreted by a radiologist with 5 years of experience and subsequently reviewed by a senior radiologist with 10 years of experience. Both readers were blinded to the clinical data. In cases of disagreement, a final consensus diagnosis was reached through discussion. In particular, at baseline and every 2–3 treatment cycles, imaging assessments included CT, bone scanning, and MRI for primary and metastatic sites.

### HER2 expression assessment

HER2 expression status was assessed by immunohistochemistry (IHC) and interpreted by experienced pathologists in strict accordance with the 2022 Chinese Expert Consensus on HER2 Testing in Urological Tumors. All interpretations were independently conducted and reviewed by two senior pathologists to ensure consistency and accuracy. Based on the IHC scoring criteria, patients were categorized into 0, 1+, 2+, and 3+ groups.

### Therapeutic response

Efficacy was assessed according to the Response Evaluation Criteria in Solid Tumors (RECIST) version 1.1. Objective response rate (ORR) is defined as the proportion of patients who achieved complete response (CR) or partial response (PR). Disease control rate (DCR) is defined as the proportion of patients who achieved CR, PR, or stable disease (SD). The primary endpoint of this study was progression-free survival (PFS), defined as the time from initiation of the combination therapy to the first radiographically confirmed disease progression or death from any cause (whichever occurred first). For surviving patients without such events by the analysis cutoff date, PFS data were censored at the date of the last valid tumor assessment. Secondary endpoints included overall survival (OS), defined as the time from treatment initiation to death from any cause, with survival time censored at the last follow-up date for patients who were still alive at the study end.

### Adverse event assessment

The incidence and severity of treatment-related adverse events (TRAEs) served as key safety endpoints in this study. All AEs were actively collected from the medical record system and rigorously graded according to the National Cancer Institute Common Terminology Criteria for Adverse Events, version 5.0. Systematic patient assessments were conducted before treatment and during each treatment cycle, including monitoring of complete blood counts, hepatic and renal function panels, coagulation parameters, and tumor markers. The protocol followed standard clinical routine practices and physician judgment.

### Statistical analysis

All statistical analyses were performed using SPSS 27.0 and R software (version 4.3.1; http://www.r-project.org/). Baseline demographic and clinicopathological characteristics, as well as adverse events, were summarized descriptively. Continuous variables are presented as median with interquartile range (IQR) and categorical variables as frequency (%). A median tumor size of 3 cm was used as the threshold. ORR and DCR with their 95% confidence intervals were calculated using the Wilson method. Survival curves were estimated by the Kaplan–Meier method, and intergroup comparisons were conducted with the log-rank test. The log-rank test was used for time-to-event endpoints, including PFS and OS. Proportional hazards testing and survival regression modeling were performed using the survival package. Visualization of results was generated with the survminer and ggplot2 packages. All tests were two-sided, and a *P*-value <0.05 was considered statistically significant.

## Results

### Baseline characteristics of the patients

This study included 132 patients with La/mUC who received RC48 in combination with a PD-1 inhibitor. Baseline demographic and clinicopathological characteristics are detailed in **Table 1**. The median age was 68 years, and there was a male predominance (72.73%). Comorbidities included hypertension (23.48%), diabetes mellitus (15.15%), and hyperlipidemia (13.64%). Among these patients, 25.76% had a baseline estimated glomerular filtration rate (eGFR) below 60 mL/min/1.73 m².

**Table 1:** Baseline characteristics of the patients.

Variables	Cases/values
Age, mean (years)	68 (61, 77)
Tumor size, median (cm)	3.0 (2.0, 4.1)
Gender, *n* (%)	
Male	96 (72.73)
Female	36 (27.27)
Histological differentiation, *n* (%)	
Urothelial carcinoma	101 (76.52)
Urothelial carcinoma with squamous differentiation	19 (14.39)
Urothelial carcinoma with glandular differentiation	12 (9.09)
Primary lesion, *n* (%)	
Bladder	94 (71.21)
Ureter/renal pelvis	38 (28.79)
Lymphovascular invasion, *n* (%)	
No	47 (35.61)
Yes	85 (64.39)
GFR, *n* (%)	
≥60	98 (74.24)
<60	34 (25.76)
Hypertension, *n* (%)	
No	101 (76.52)
Yes	31 (23.48)
Diabetes, *n* (%)	
No	112 (84.85)
Yes	20 (15.15)
Hyperlipidemia, *n* (%)	
No	114 (86.36)
Yes	18 (13.64)
Metastatic site, *n* (%)	
No	52 (39.40)
Lymph node	48 (60.00)
Lung	29 (36.25)
Liver	25 (31.25)
Bone	23 (28.75)
Others	13 (16.25)
Drug type, *n* (%)	
RC48 + toripalimab	72 (54.55)
RC48 + tislelizumab	60 (45.45)
Surgery, *n* (%)	
Radical surgery	60 (45.45)
Partial resection surgery	24 (18.18)
Diagnostic surgery	35 (26.52)
Tumor biopsy	13 (9.85)
Previous treatment, *n* (%)	
No	95 (71.97)
Chemotherapy/radiotherapy	37 (28.03)
HER2 expression, *n* (%)	
0+	23 (17.42)
1+	35 (26.52)
2+	45 (34.09)
3+	29 (21.97)

Metastatic site: others include adrenal, brain metastasis, abdominal metastasis, etc.

The primary tumors were predominantly bladder cancer (71.21%), with the vast majority histologically pure urothelial carcinoma (76.52%). Cases with squamous or glandular differentiation accounted for 14.39% and 9.09%, respectively. Notable high-risk pathological features included lymphovascular invasion (LVI), present in up to 64.39% of patients. Regarding HER2 expression status, patients showed a wide distribution: IHC scores of 0, 1+, 2+, and 3+ accounted for 17.42%, 26.52%, 34.09%, and 21.97%, respectively, with HER2-high expression (2+/3+) comprising 56.06% in total. Distant metastases were present in 80 patients (60.61%), with the most common sites being the lymph nodes (60.00%), lungs (36.25%), liver (31.25%), and bones (28.75%), in descending order. Local interventions included radical surgery (45.45%), partial resection (18.18%), diagnostic surgery (26.52%), and tumor biopsy (9.85%). RC48 was administered as first-line treatment in 95 patients (71.97%), while 37 patients had previously undergone radiotherapy or chemotherapy. All patients received combination regimens, specifically RC48 plus toripalimab (54.55%) or tislelizumab (45.45%).

### Therapeutic effect

**Figure 1:**
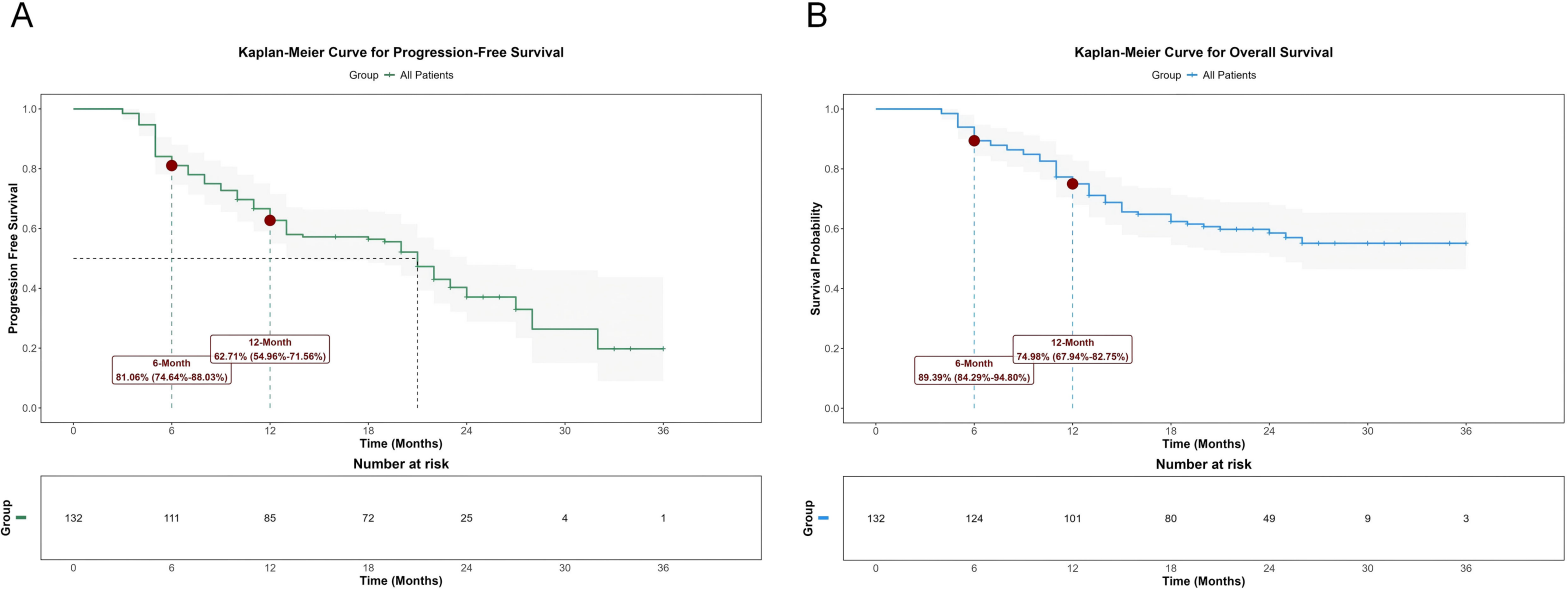
Analysis of PFS **(A)** and OS **(B)** in all patients.

The median follow-up duration was 25 months (range: 4–36 months). Antitumor efficacy was assessed according to RECIST version 1.1. Among all 132 patients, 26 (19.70%) achieved CR, 68 (51.52%) achieved PR, and 23 (17.42%) had SD. Based on these results, the ORR was 71.21% (95% CI: 62.97%–78.25%), and the DCR was 88.64% (95% CI: 82.10%–92.99%). The median PFS was 21 months (95% CI: 14–24 months), and the median OS was not reached. The number and proportion of observed OS events, specifically 55 events, accounted for 41.67% of the cohort. The 6-month PFS rate was 81.06% (95% CI: 74.64%–88.03%), and the 12-month PFS rate was 62.71% (95% CI: 54.96%–71.56%). The 6-month OS rate was 89.39% (95% CI: 84.29%–94.80%), and the 12-month OS rate was 74.98% (95% CI: 67.94%–82.75%) (**Figure 1**).

In-depth subgroup analysis demonstrated that the RC48 plus PD-1 inhibitor regimen maintained favorable efficacy across patients with different underlying conditions. There was no significant difference in ORR between patients without hypertension and those with hypertension: 73.27% (95% CI: 63.90%–80.93%) vs. 64.52% (95% CI: 46.95%–78.88%), *P* = 0.370. Diabetes (*P* = 0.593), renal dysfunction (eGFR < 60) (*P* = 0.662), and hyperlipidemia (*P* = 0.588) did not significantly affect treatment response (**Table 2**). The multivariate Cox regression analyses of all variables are presented in **Supplementary Tables S4**, **S5**.

**Table 2:** Subgroup analysis of comorbid populations.

Subgroup analysis of comorbid populations (ORR)									
Efficacy evaluation	Overall (*n* = 132)	Comorbid condition							
		Hypertension		Diabetes		eGFR		Hyperlipidemia	
		No (*n* = 101)	Yes (*n* = 31)	No (*n* = 112)	Yes (*n* = 20)	<60 (*n* = 34)	≥60 (*n* = 98)	No (*n* = 114)	Yes (*n* = 18)
ORR, % (95% CI)	71.21% (62.97%–78.25%)	73.27% (63.90%–80.93%)	64.52% (46.95%–78.88%)	72.32% (63.40%–79.76%)	65.00% (43.29%–81.88%)	72.45% (62.88%–80.32%)	67.65% (50.84%–80.87%)	70.18% (61.23%–77.80%)	77.78% (54.79%–91.00%)
*P*‐value	–	0.37		0.593		0.662		0.588	

Among other key prognostic factors, patients with pure urothelial carcinoma exhibited the highest ORR at 77.23% (95% CI: 68.14–84.32). The ORR decreased to 52.63% (95% CI: 31.71–72.67) in tumors with squamous differentiation and reached the lowest level of 50.00% (95% CI: 25.38–74.62) in those with glandular differentiation. The difference in ORR among the various histological subtypes was statistically significant (*P* = 0.022), indicating that variant differentiation is a negative predictive factor for treatment efficacy. The presence of lymphovascular invasion significantly affected treatment efficacy [63.53% (95% CI: 52.92%–72.97%) vs. 85.11% (95% CI: 72.31%–92.59%), *P* = 0.009], with patients having a maximum tumor diameter ≤3 cm demonstrating superior ORR [81.25% (95% CI: 70.03%–88.94%) vs. 61.76% (95% CI: 49.88%–72.39%), *P* = 0.020]. Additionally, treatment response showed a clear hierarchical correlation with HER2 expression levels. The ORR was lower in HER2 0/1+ patients [47.83% (95% CI: 29.24%–67.04%)] and HER2 1+ patients [65.71% (95% CI: 49.15%–79.17%)]. HER2 2+ patients showed a significantly increased ORR of 80.00% (95% CI: 66.18%–89.10%), and the highest ORR was observed in HER2 3+ patients at 82.76% (95% CI: 65.45%–92.40%). The difference in ORR among groups with different HER2 expression levels was statistically significant (*P* = 0.017), confirming that high HER2 expression (2+ and 3+) may serve as a strong biomarker for predicting higher ORR. The treatment response was not influenced by the primary tumor site (*P* = 0.675), drug type (*P* = 0.249), previous other treatments (chemotherapy/radiotherapy) (*P* = 0.392), or surgery (*P* = 0.767). See **Supplementary Tables S1**, **S2**.

The study found that the presence of distant metastasis at baseline is a key factor affecting ORR. Compared with patients without corresponding metastatic lesions, those with lymph node metastasis had an ORR of 58.33% (95% CI: 44.28%–71.15%) vs. 78.57% (95% CI: 68.65%–85.99%), *P* = 0.017; lung metastasis 51.72% (95% CI: 34.43%–68.61%) vs. 76.70% (95% CI: 67.67%–83.81%), *P* = 0.012; liver metastasis 52.00% (95% CI: 33.50%–69.97%) vs. 75.70% (95% CI: 66.78%–82.84%), *P* = 0.027; and bone metastasis 52.17% (95% CI: 32.96%–70.76%) vs. 75.23% (95% CI: 66.36%–82.38%), *P* = 0.041. Each comparison showed a substantial decline of approximately 20 to 25 percentage points in ORR, with statistically significant differences, indicating that visceral and bone metastases are definitive unfavorable prognostic indicators (**Table 3**).

**Table 3:** Subgroup analysis of patients with distant metastasis.

Analysis of clinical and pathological subgroup populations (ORR)									
Efficacy evaluation	Overall (*n* = 132)	Metastatic site							
		Lymph node		Lung		Liver		Bone	
		No (*n* = 84)	Yes (*n* = 48)	No (*n* = 103)	Yes (*n* = 29)	No (*n* = 107)	Yes (*n* = 25)	No (*n* = 1,109)	Yes (*n* = 23)
ORR, % (95% CI)	71.21% (62.97%–78.25%)	78.57% (68.65%–85.99%)	58.33% (44.28%–71.15%)	76.70% (67.67%–83.81%)	51.72% (34.43%–68.61%)	75.70% (66.78%–82.84%)	52.00% (33.50%–69.97%)	75.23% (66.36%–82.38%)	52.17% (32.96%–70.76%)
*P*‐value	–	0.017		0.012		0.027		0.041	

**Figure 2:**
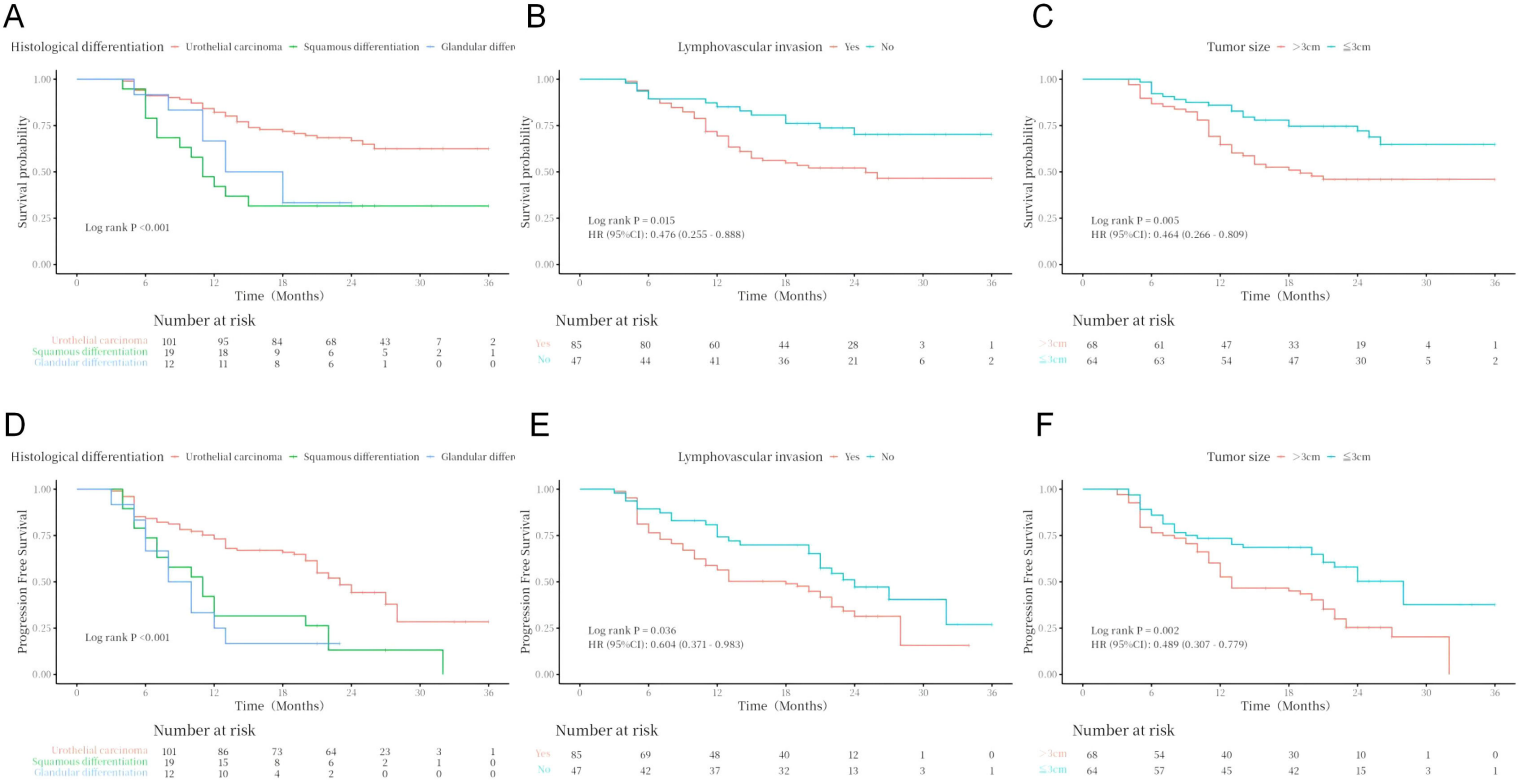
Analysis of OS (**A–C**) and PFS (**D–F**) by histologic variant type, lymphovascular invasion status, and tumor size.

**Figure 3:**
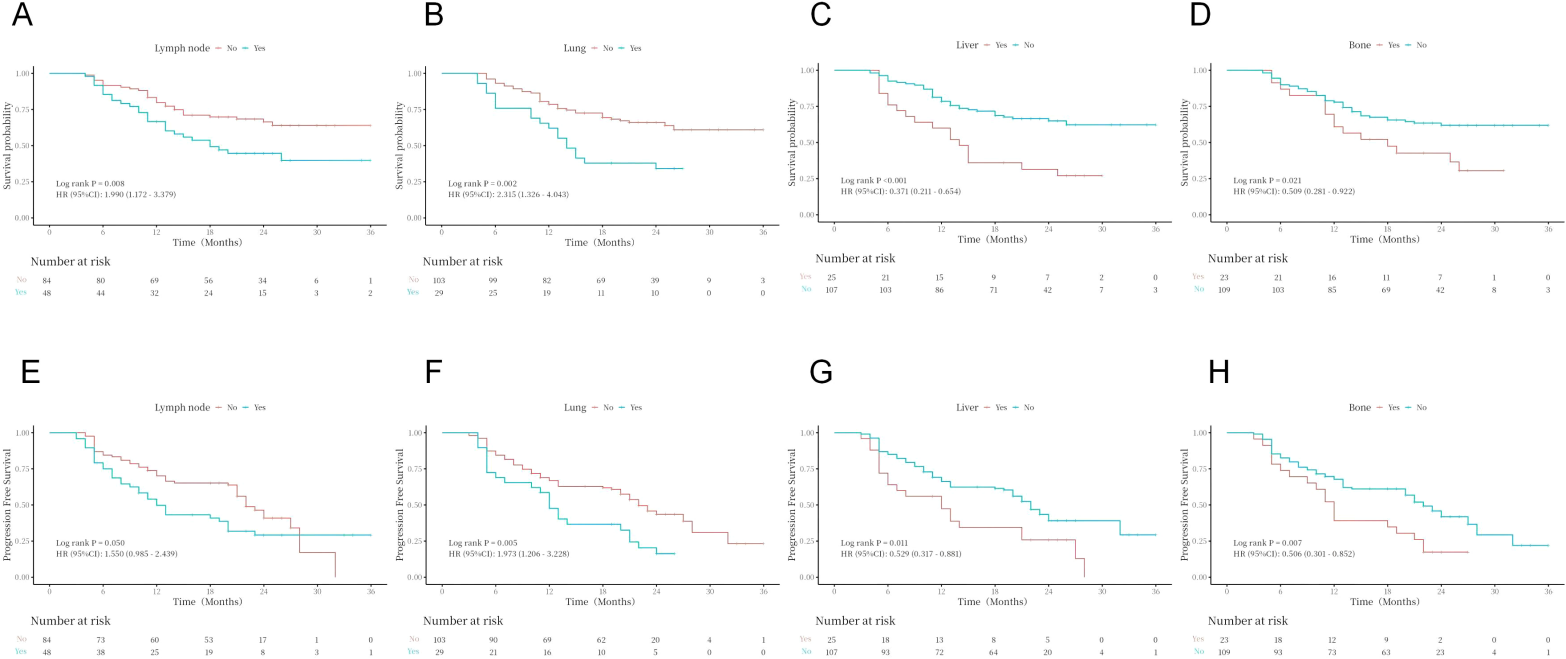
Analysis of OS (**A–D**) and PFS (**E–H**) in different metastatic organs.

**Figure 4:**
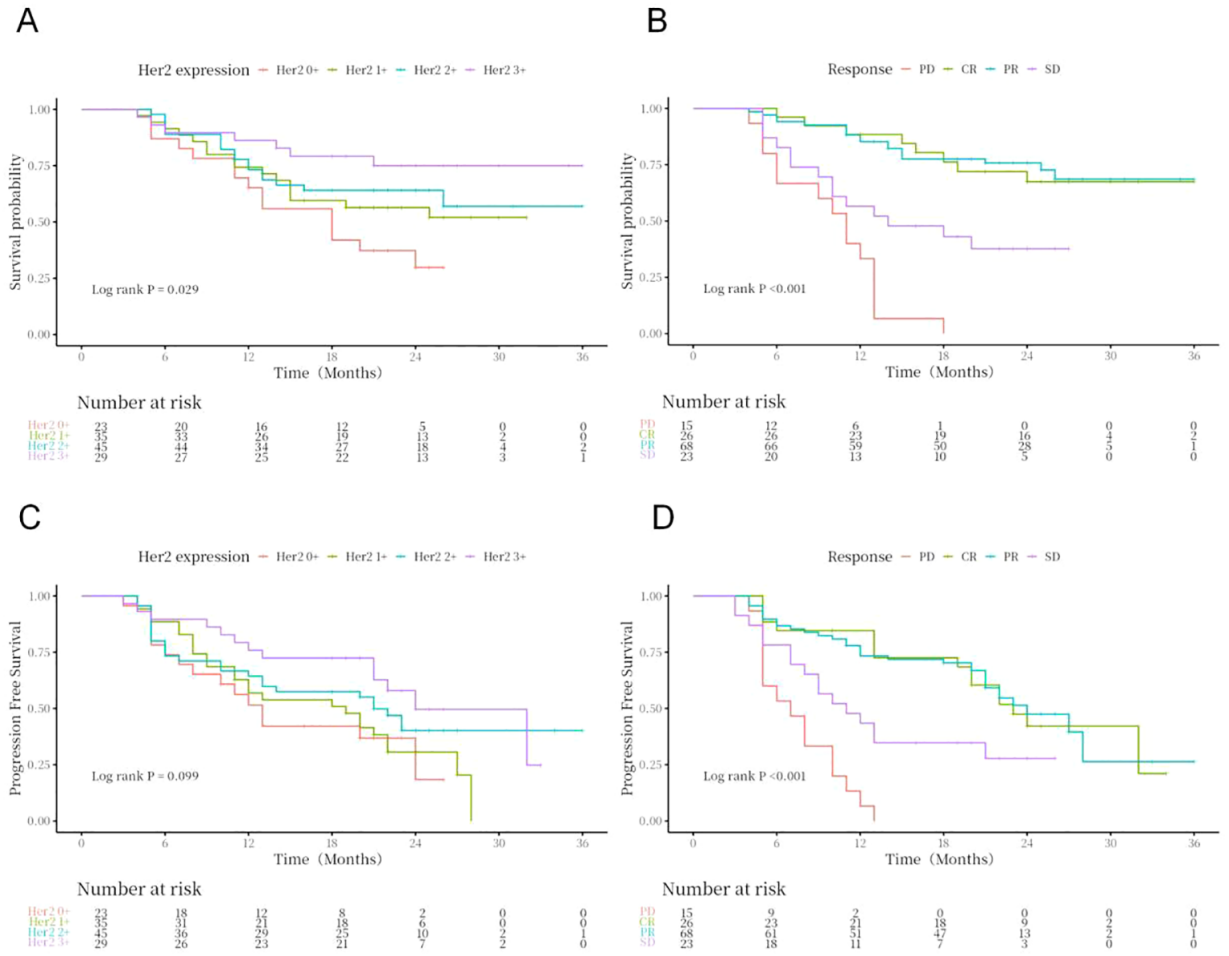
OS **(A, B)** and PFS **(C, D)** stratified by HER2 expression and treatment response.

**Figure 5:**
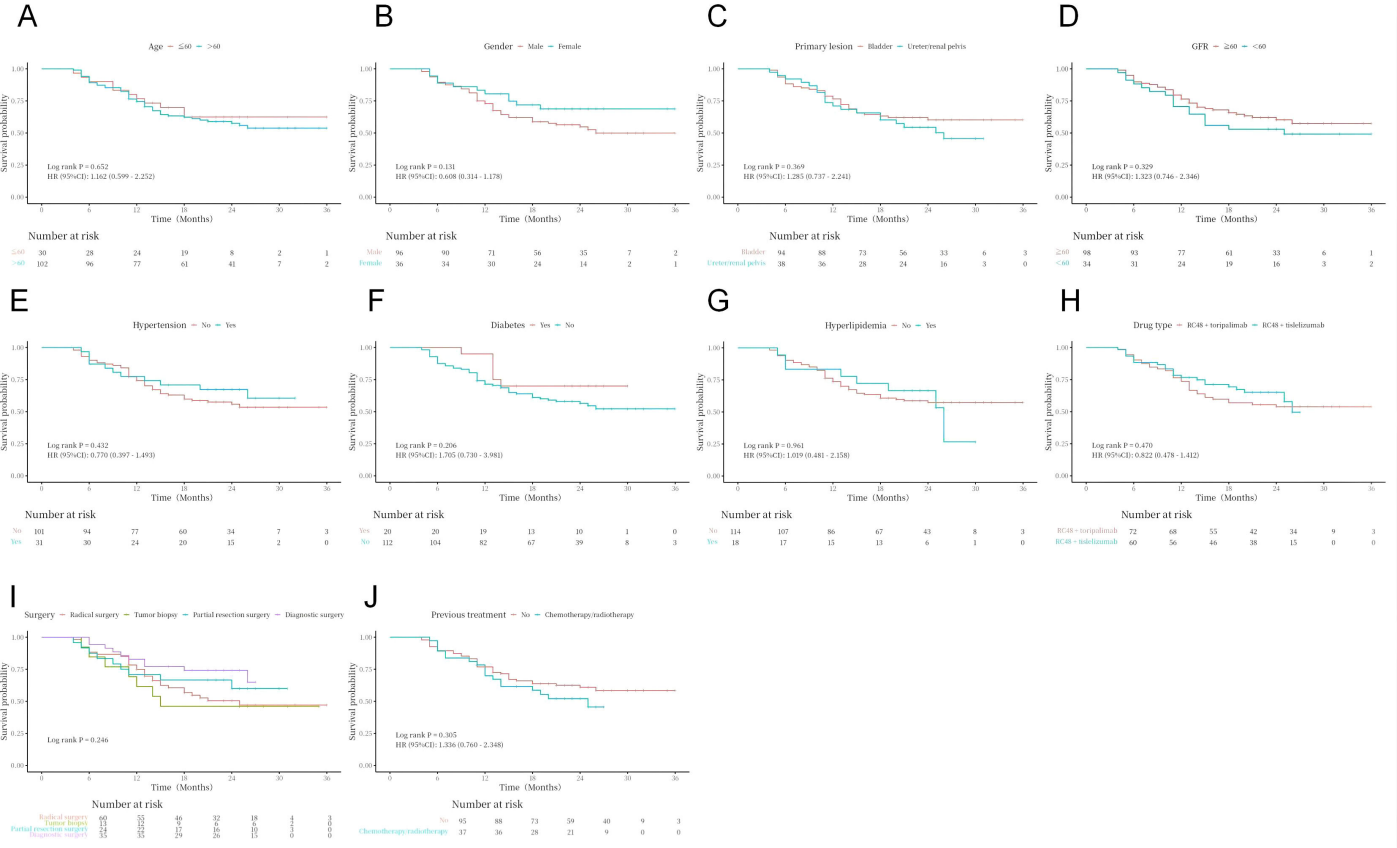
Analysis of OS (**A–J**) in relation to clinical variable characteristics and comorbidity features.

Kaplan–Meier curves and log-rank test analyses of PFS and OS for key clinical and pathological variables revealed statistically significant effects on patient prognosis after subgroup stratification. Specifically, histological variant type (*P* < 0.001), lymphovascular invasion status (*P* = 0.036), tumor size (*P* = 0.002), baseline metastatic status (lymph node, *P* = 0.050; lung, *P* = 0.005; liver, *P* = 0.011; bone, *P* = 0.007), and treatment response (*P* < 0.001) were all significant prognostic factors for PFS (**Figures 2**–**4**). Similarly, these variables, along with HER2 expression level (*P* = 0.029), were confirmed as independent prognostic predictors for OS. Notably, although HER2 expression levels did not show significant stratification effects on PFS in this cohort, their strong correlation with OS suggests that their impact may be more pronounced in long-term survival outcomes (**Figure 4**). Conversely, variables such as age, gender, primary site, common comorbidities (hypertension, diabetes, renal function, hyperlipidemia), drug combination types, prior treatment history, and surgical approaches did not demonstrate significant prognostic value for either PFS or OS in this analysis (**Figure 5**; **Supplementary Figure S1**).

### Safety

At the data cutoff for this study, all enrolled patients had completed safety assessments and survival follow-up. Among the overall study population, the median number of RC48 treatment cycles was 10 (range: 4–20 cycles), while the median number of PD−1 inhibitor treatment cycles was 8 (range: 2–16 cycles). The median treatment duration was 5.8 months (range: 1.5–18.5 months). The overall incidence of TRAEs was 74.24%, with most events being grade 1–2 and well-tolerated. A total of 18 patients (13.64%) experienced grade 3 TRAEs, which were effectively managed through dose adjustments or temporary discontinuation; no grade 4 or 5 TRAEs were observed. TRAEs with higher incidence included fatigue (38.64%), nausea (35.61%), anemia (32.58%), pruritus (28.79%), and peripheral neuropathy (23.48%). Grade 3 TRAEs primarily consisted of anemia, peripheral neuropathy, elevated serum creatinine, elevated transaminases, and neutropenia (**Table 4**). Among the 63 treatment discontinuations, poor efficacy was the primary reason (39.68%), followed by financial reasons (17.46%), adverse reactions (15.87%), and unknown causes (12.69%). Additionally, 9 patients (14.29%) discontinued treatment due to achieving an excellent CR. No treatment-related deaths occurred during the therapy period.

**Table 4:** Adverse events of patients’ treatment.

Adverse event, *n* (%)	Patients (*n* = 132) any grade	Grade ≥3
Fatigue	51 (38.64%)	0
Nausea	47 (35.61%)	0
Anemia	43 (32.58%)	6 (4.55%)
Pruritus	38 (28.79%)	0
Peripheral neuropathy	31 (23.48%)	3 (2.27%)
Elevated serum creatinine	27 (20.45%)	2 (1.52%)
Hypoalbuminemia	25 (18.94%)	0
Leukopenia	22 (16.67%)	0
Elevated transaminases	20 (15.15%)	4 (3.03%)
Neutropenia	19 (14.40%)	3 (2.27%)
Hyperglycemia	16 (12.12%)	0
Constipation	13 (9.85%)	0
Weight loss	13 (9.85%)	0
Diarrhea	10 (7.58%)	0
Thyroid dysfunction	5 (3.79%)	0

## Discussion

RC48 is a novel HER2-targeting ADC composed of a humanized anti-HER2 monoclonal antibody conjugated to the microtubule inhibitor MMAE via a cleavable linker (15). Its mechanism of action involves precise recognition and internalization into HER2-expressing tumor cells, followed by the release of the cytotoxic payload to disrupt the microtubule system and induce tumor cell apoptosis (16, 17). RC48 has been approved for the treatment of HER2-overexpressing La/mUC (18). ADCs, due to their unique “targeted delivery” mechanism, can enhance antitumor efficacy while relatively reducing systemic toxicity (19). In recent years, combination strategies of ADCs with immune checkpoint inhibitors (such as PD-1 inhibitors) have attracted widespread attention. The theoretical basis is that ADC-induced tumor cell death can release tumor-associated antigens and potentially trigger immunogenic cell death, thereby altering the tumor microenvironment and enhancing T-cell infiltration and function. This synergizes with the immune-releasing effect of PD-1 inhibitors, promising cooperative antitumor efficacy (20, 21).

The efficacy of RC48 in La/mUC was initially established through monotherapy clinical trials (22). A pivotal single-arm phase II trial (NCT03507166) enrolled 43 previously treated HER2-overexpressing (IHC 2+ or 3+) mUC patients. The independently assessed ORR was 51.2% (95% CI: 35.5%–66.7%), with median PFS and OS of 6.9 months (95% CI: 5.6–8.9) and 13.9 months (95% CI: 9.1–NE), respectively, confirming its significant single-agent activity (23). Subsequently, an exploratory phase I/II study (NCT04073602) evaluated RC48 in HER2-low (IHC 0+/1+) La/mUC patients, demonstrating an ORR of 31.6% (95% CI: 12.6–56.6), with median PFS and OS of 5.5 months (95% CI: 3.9–5.7) and 16.4 months (6.8–26.8 months), respectively, indicating its antitumor activity in HER2-low La/mUC patients (24). Regarding combination therapy, a multicenter retrospective real-world study evaluated the safety and efficacy of RC48 combined with PD-1 inhibitors as neoadjuvant therapy for locally muscle-invasive bladder cancer, supporting RC48–PD-1 combinations as a neoadjuvant strategy for localized MIBC (25). These findings provide high-level evidence for the real-world application of RC48 in combination with PD-1 inhibitors.

Locally advanced or metastatic urothelial carcinoma predominantly affects elderly patients who often have multiple comorbidities such as hypertension, diabetes, chronic renal insufficiency, and hyperlipidemia (26, 27). These comorbidities may not only affect patients’ performance status and tolerance to treatment but also limit the use of standard cisplatin-based regimens due to factors such as renal impairment (28). Therefore, evaluating the efficacy and safety of RC48–PD-1 inhibitors in this substantial real-world population is crucial. Theoretically, comorbidities may influence treatment effectiveness by altering drug metabolism, increasing the risk of organ-specific toxicity, or changing tumor biology (29, 30). Additionally, polypharmacy and frailty may lead to higher rates of treatment-related adverse events (31). Systematically analyzing the impact of comorbidities on combination therapy holds significant clinical importance for personalized therapy and optimized patient management.

Beyond baseline patient characteristics, various clinical and pathological factors may also influence RC48–PD-1 inhibitor outcomes. Tumor-burden-related factors, such as primary tumor size, presence of lymphovascular invasion, and sites of distant metastasis (e.g., liver, lung, or bone metastases), are established prognostic indicators. Intrinsic tumor biology, including histological subtype (pure urothelial carcinoma vs. variant differentiation) and HER2 expression levels, may directly determine ADC targeting efficiency and therapeutic efficacy. Systematically evaluating the impact of these variables on ORR, PFS, and OS helps identify populations with superior benefits, provide early warning for potential poor responders, and support more refined treatment decision-making.

This multicenter real-world study analyzed 132 patients with La/mUC over a median follow-up of 25 months. The combination of RC48 and PD-1 inhibitors yielded an ORR of 71.21% and a DCR of 88.64%. Median PFS was 21 months, while median OS was not reached, underscoring the substantial antitumor activity of this regimen in real-world practice. This observed ORR surpasses historical PD-1 monotherapy data, likely due to RC48’s synergistic induction of immunogenic cell death and efficacy in HER2 1+ populations. Subgroup analyses identified key prognostic determinants. Comorbidity assessments showed that the presence of hypertension, diabetes, or renal insufficiency (eGFR < 60 mL/min) did not significantly affect ORR (*P* > 0.05), indicating consistent efficacy across diverse patient subgroups. Tumor histology also influenced outcomes: patients with variant histology (predominantly squamous or glandular differentiation) exhibited significantly lower ORR than those with pure urothelial carcinoma (*P* = 0.022). Additionally, lymphovascular invasion (*P* = 0.009) and maximum tumor diameter >3 cm (*P* = 0.020) were associated with inferior response. Notably, HER2 expression levels demonstrated a clear positive correlation with ORR (IHC 3+ > 2+ > 1+/0; *P* = 0.017), confirming high HER2 expression as a robust predictive biomarker. Baseline metastases to lymph nodes, lung, liver, or bone were independently associated with significantly reduced ORR, highlighting the negative impact of high tumor burden and visceral involvement (all *P* < 0.05). Survival analyses reinforced these findings. Histological variants, lymphovascular invasion, tumor size, baseline metastatic sites, and early treatment response were significantly associated with PFS (*P* < 0.05). These factors, along with HER2 expression levels, also correlated with OS (*P* < 0.05) and were confirmed as independent prognostic factors for OS. In contrast, age, sex, primary tumor site, specific PD-1 inhibitor used (toripalimab vs. tislelizumab), and prior treatment history did not demonstrate significant prognostic value. Regarding safety, TRAEs occurred in 74.24% of patients, the majority being grade 1–2 and clinically manageable. Grade 3 TRAEs were observed in 13.64% of cases, primarily hematologic toxicity and peripheral neuropathy. No grade 4 or 5 events or treatment-related deaths were reported, confirming an acceptable safety profile for this combination in an expanded real-world population.

This study has several limitations. First, its retrospective design may introduce selection and information biases. The absence of a concurrent control group precludes direct comparisons with standard therapies and causal inference, and selection bias may influence the observed efficacy. Second, despite a relatively large real-world cohort, statistical power may be limited for certain subgroup analyses. The large number of subgroup analyses performed without adjustment for multiple comparisons increases the risk of false-positive findings. Therefore, these analyses should be interpreted as exploratory with *P*-values provided for reference only. Third, some prognostic factors identified in univariate analyses did not retain independent significance in multivariate Cox regression analyses, requiring further validation in larger prospective cohorts. Fourth, HER2 status was primarily assessed by immunohistochemistry without routine confirmation by fluorescence *in situ* hybridization or next-generation sequencing, limiting the ability to assess gene amplification or identify additional biomarkers. Fifth, the median follow-up remains insufficient for comprehensive long-term survival evaluation, particularly for overall survival, which has not yet been reached. Thus, the overall survival findings should be considered preliminary. Additionally, due to the combined use of RC48 and PD-1 inhibitors, distinguishing RC48-related adverse events from PD-1-related immune-related adverse events presents inherent challenges in causality attribution. Furthermore, post-progression treatment data were not systematically collected, which may affect overall survival interpretation.

Future prospective studies are warranted to address these limitations. Well-designed prospective investigations should incorporate systematic collection of detailed comorbidity data to evaluate the impact of hypertension, diabetes, and renal insufficiency on treatment outcomes across different patient subgroups. Comparative analyses between RC48 plus PD-1 inhibitor combination therapy and each monotherapy are needed to enable systematic distinction of adverse event profiles associated with each agent. Comprehensive documentation of post-progression treatment information should be prioritized to facilitate accurate assessment of overall survival. Additionally, future prospective multicenter randomized controlled trials incorporating standardized biomarker testing, including HER2 immunohistochemistry and fluorescence *in situ* hybridization, as well as next-generation sequencing in central laboratories, are warranted to further validate the therapeutic benefits of this combination and establish a precision efficacy prediction model.

## Conclusion

The combination of RC48 and PD-1 inhibitors exhibits robust antitumor activity and a manageable safety profile in real-world practice, reinforcing its therapeutic potential beyond clinical trials. Importantly, its efficacy remains consistent across patients with common comorbidities, while its prognostic impact is significantly influenced by specific tumor characteristics such as histological variants, lymphovascular invasion, and HER2 expression, which may provide valuable insights for treatment optimization.

## Data Availability

The original contributions presented in the study are included in the article/**Supplementary Material**. Further inquiries can be directed to the corresponding author.
