# Supplementary material for: Disitamab vedotin (RC48) combined with PD-1 inhibitors in locally advanced or metastatic urothelial carcinoma: clinical outcomes and prognostic factors from a multicenter real-world study
Source: Front Immunol. 2026 Apr 29;17:1815591. doi: 10.3389/fimmu.2026.1815591 (PMC13168194; doi:10.3389/fimmu.2026.1815591)
Supplement: Supplementary file 1 [file Table1.docx]

**Table S1 Subgroup analysis based on tumor characteristics**

| **Analysis of Clinical and Pathological Subgroup Populations (ORR)** | | | | | | | | | | |
| --- | --- | --- | --- | --- | --- | --- | --- | --- | --- | --- |
| Efficacy  evaluation | Overall  (n= 132) | Grouping variable | | | | | | | | |
|  |  | Primary lesion | | Histological differentiation | | | Lymphovascular invasion | | Tumor size | |
|  |  | Bladder (n=94) | Ureter/renal pelvis (n=38) | Urothelial carcinoma(n=101) | Squamous differentiation(n=19) | Glandular differentiation(n=12) | No(n=47) | Yes(n=85) | ≤3cm(n=64) | >3cm(n=68) |
| ORR, %(95% CI) | 71.21% (62.97% - 78.25%) | 72.34% (62.56% - 80.37%) | 68.42% (52.54% - 80.92%) | 77.23% (68.14% - 84.32%) | 52.63% (31.71% - 72.67%) | 50.00% (25.38% - 74.62%) | 85.11% (72.31% - 92.59%) | 63.53% (52.92% - 72.97%) | 81.25% (70.03% - 88.94%) | 61.76% (49.88% - 72.39%) |
|  |  |  |  |  |  |  |  |  |  |  |
|  |  |  |  |  |  |  |  |  |  |  |
| P‐value | - | 0.675 | | 0.022 | | | 0.009 | | 0.02 | |

**Table S2 Subgroup analysis based on treatment information**

| **Analysis of Clinical and Pathological Subgroup Populations (ORR)** | | | | | | | | | | | | | |
| --- | --- | --- | --- | --- | --- | --- | --- | --- | --- | --- | --- | --- | --- |
| Efficacy  evaluation | Overall  (n= 132) | Grouping variable | | | | | | | | | | | |
|  |  | Drug type | | Her2 expression | | | | Previous treatment | | Previous surgery | | | |
|  |  | RC48 + toripalimab (n=72) | RC48 + tislelizumab (n=60) | 0+  (n=23) | 1+ (n=35) | 2+ (n=45) | 3+ (n=29) | No(n=95) | Chemotherapy/radiotherapy(n=37) | Radical surgery (n=60) | Partial resection surgery(n=24) | Diagnostic surgery (n=35) | Tumor biopsy (n=13) |
| ORR, %(95% CI) | 71.21% (62.97% - 78.25%) | 66.67% (55.18% - 76.47%) | 76.67% (64.56% - 85.56%) | 47.83% (29.24% - 67.04%) | 65.71% (49.15% - 79.17%) | 80.00% (66.18% - 89.10%) | 82.76% (65.45% - 92.40%) | 73.68% (64.03% - 81.49%) | 64.86% (48.76% - 78.17%) | 66.67% (54.06% - 77.27%) | 75.00% (55.10% - 88.00%) | 74.29% (57.93% - 85.84%) | 76.92% (49.74% - 91.82%) |
|  |  |  |  |  |  |  |  |  |  |  |  |  |  |
|  |  |  |  |  |  |  |  |  |  |  |  |  |  |
| P‐value | - | 0.249 | | 0.017 | | | | 0.392 | | 0.767 | | | |

**Table S3 Summary of Key Real-World Study Comparisons for RC48 Combined with PD-1 Inhibitors in Urothelial Carcinoma**

| Year | Study | Authors | Key Characteristics and Focus |
| --- | --- | --- | --- |
| February 2025 | The effectiveness and safety of RC48 alone or in combination with PD-1 inhibitors for locally advanced or metastatic urothelial carcinoma: a multicenter, real-world study | Ge H, Liu C, Shen C, et al. | Multicenter study (n=42); evaluated efficacy of RC48 monotherapy or combined with PD-1 inhibitors; focused on efficacy differences across treatment lines |
| September 2025 | The efficacy and safety of disitamab vedotin combined with immune checkpoint inhibitors in metastatic upper tract urothelial carcinoma: a multicenter real-world study | Ng C, Jing T, Yu S, et al | Multicenter study (n=198); evaluated efficacy of RC48-ADC plus ICIs in metastatic upper tract urothelial carcinoma; focused on first-line versus later-line treatment outcomes |
| July 2023 | HER2-targeting antibody-drug conjugate RC48 alone or in combination with immunotherapy for locally advanced or metastatic urothelial carcinoma: a multicenter, real-world study | Chen M, Yao K, Cao M, et al | Multicenter study (n=36); evaluated RC48 monotherapy versus RC48 combined with PD-1 inhibitors; focused on efficacy comparison between combination and monotherapy regimens |
| December 2025 | RC48-ADC monotherapy or in combination with immunotherapy for locally advanced or metastatic urothelial carcinoma with HER2 low and null expression: a multicenter, real-world, retrospective study | Wang D, Cao M, Zhang Y, et al | Multicenter study (n=27); focused on HER2 low-expression and null-expression populations; demonstrated that HER2 low-expression patients still benefit from RC48 therapy |
| December 2023 | Real-world effectiveness and safety of RC48-ADC alone or in combination with PD-1 inhibitors for patients with locally advanced or metastatic urothelial carcinoma: A multicenter, retrospective clinical study | Xu J, Zhang H, Zhang L, et al | Single-center study (n=38); evaluated RC48 monotherapy or combined with PD-1 inhibitors; focused on efficacy and safety in HER2-positive and HER2-negative populations after prior chemotherapy failure |

**A B**

**
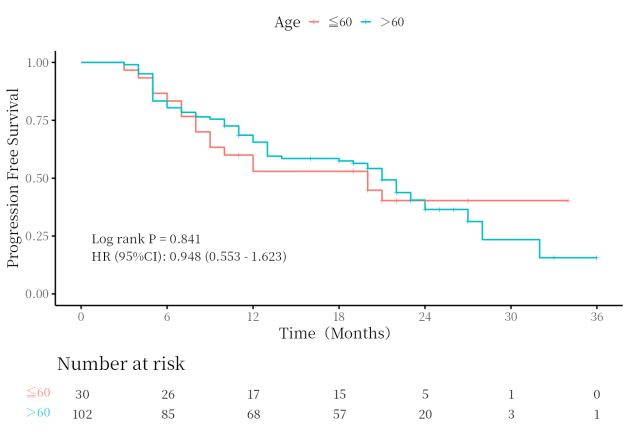

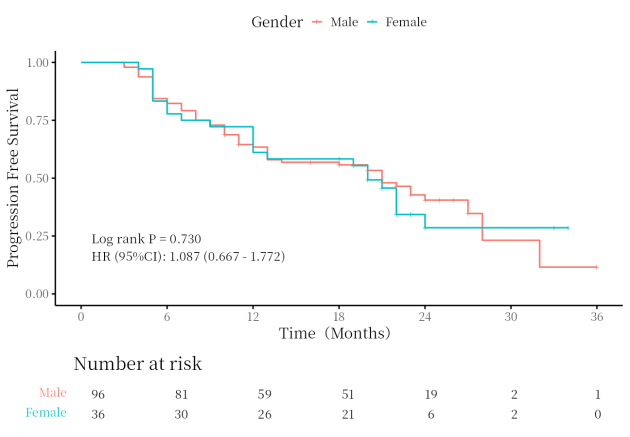
**

**C D**

**
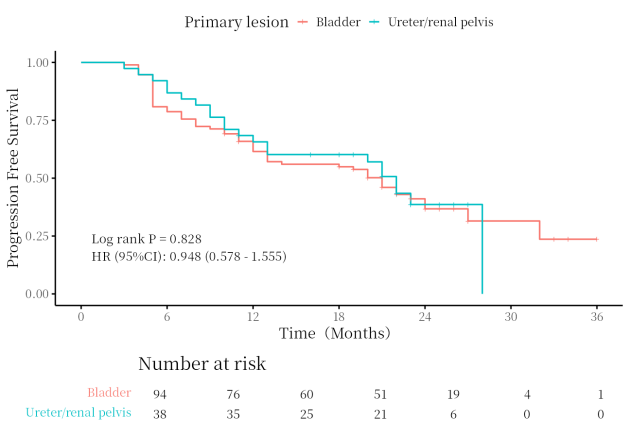

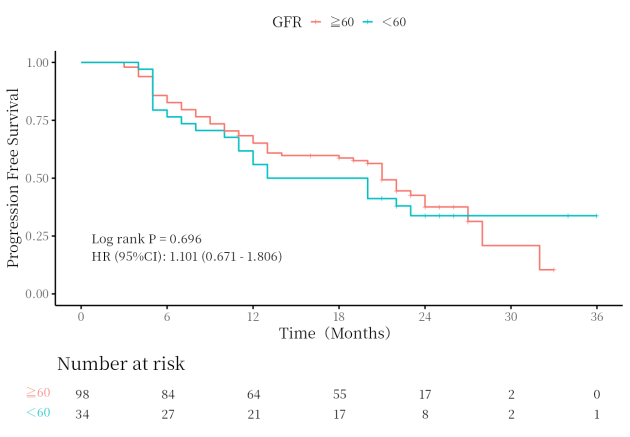
**

**E F**

**
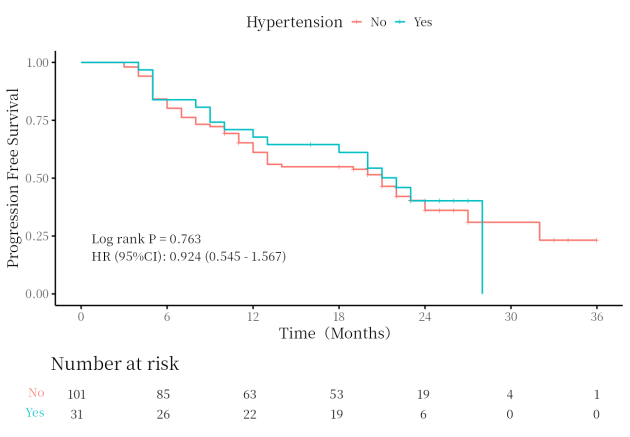

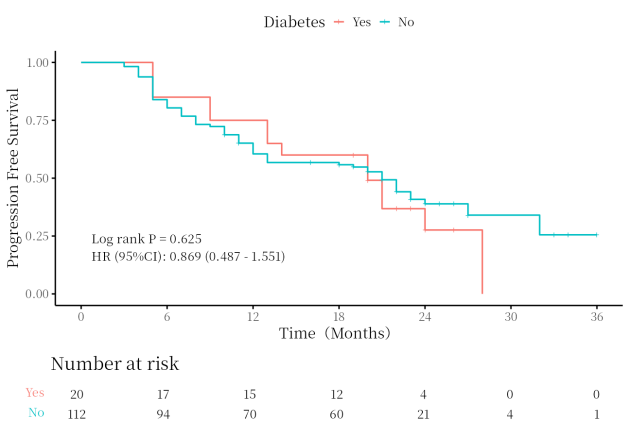
**

**G H**

**
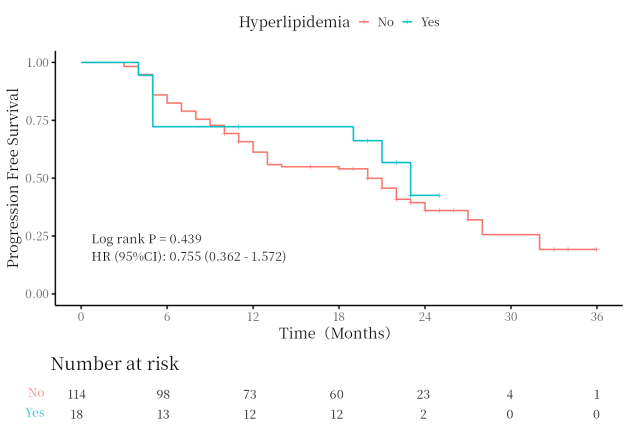

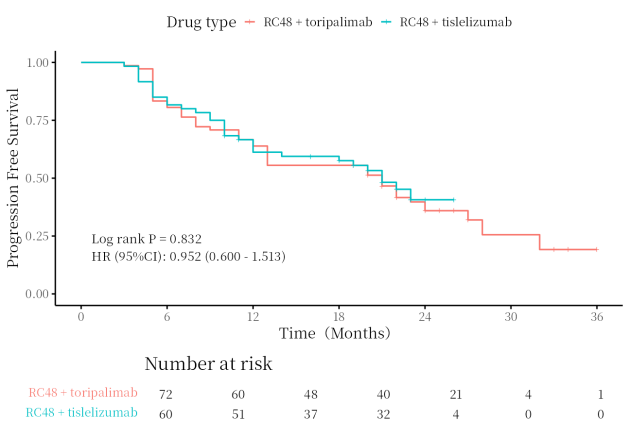
**

**I J**

**
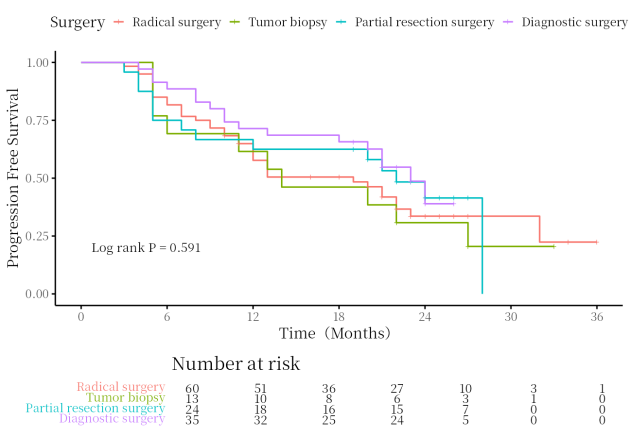

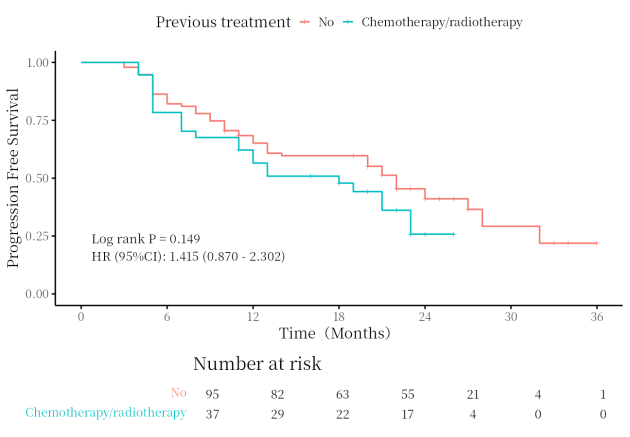
**

**Figure S1 Analysis of PFS (A-J) in relation to clinical variable characteristics and comorbidity features**

**Table S4 OS-based multifactorial Cox regression analysis**

| Variables | β | S.E | Z | *P* | HR (95%CI) |
| --- | --- | --- | --- | --- | --- |
|  |  |  |  |  |  |
| Age |  |  |  |  |  |
| ≤60 |  |  |  |  | Reference |
| >60 | -0.07 | 0.46 | -0.15 | 0.881 | 0.93 (0.38 ~ 2.30) |
| Gender |  |  |  |  |  |
| Male |  |  |  |  | Reference |
| Female | -0.15 | 0.51 | -0.29 | 0.775 | 0.86 (0.32 ~ 2.36) |
| Primary lesion |  |  |  |  |  |
| Bladder |  |  |  |  | Reference |
| Ureter/renal pelvis | 0.38 | 0.41 | 0.93 | 0.353 | 1.46 (0.66 ~ 3.26) |
| Histological differentiation |  |  |  |  |  |
| Urothelial carcinoma |  |  |  |  | Reference |
| Urothelial carcinoma with squamous differentiation | 0.90 | 0.49 | 1.83 | 0.067 | 2.47 (0.94 ~ 6.48) |
| Urothelial carcinoma with glandular differentiation | 1.56 | 0.54 | 2.90 | 0.004 | 4.74 (1.66 ~ 13.55) |
| Lymphovascular invasion |  |  |  |  |  |
| No |  |  |  |  | Reference |
| Yes | 2.07 | 0.54 | 3.85 | <0.001 | 7.90 (2.76 ~ 22.62) |
| Tumor size |  |  |  |  |  |
| ≤3cm |  |  |  |  | Reference |
| >3cm | 1.37 | 0.52 | 2.61 | 0.009 | 3.92 (1.41 ~ 10.92) |
| Hypertension |  |  |  |  |  |
| No |  |  |  |  | Reference |
| Yes | -0.19 | 0.48 | -0.39 | 0.694 | 0.83 (0.33 ~ 2.11) |
| Diabetes |  |  |  |  |  |
| No |  |  |  |  | Reference |
| Yes | -0.09 | 0.47 | -0.19 | 0.850 | 0.91 (0.36 ~ 2.30) |
| GFR |  |  |  |  |  |
| ≥60 |  |  |  |  | Reference |
| <60 | -0.01 | 0.43 | -0.01 | 0.991 | 1.00 (0.43 ~ 2.32) |
| Hyperlipidemia |  |  |  |  |  |
| No |  |  |  |  | Reference |
| Yes | -0.64 | 0.57 | -1.12 | 0.261 | 0.53 (0.17 ~ 1.61) |
| Drug type |  |  |  |  |  |
| RC48 + toripalimab |  |  |  |  | Reference |
| RC48 + tislelizumab | 0.60 | 0.40 | 1.49 | 0.137 | 1.82 (0.83 ~ 4.02) |
| Lymph node |  |  |  |  |  |
| No |  |  |  |  | Reference |
| Yes | 1.06 | 0.54 | 1.98 | 0.047 | 2.90 (1.01 ~ 8.30) |
| Lung |  |  |  |  |  |
| No |  |  |  |  | Reference |
| Yes | 0.12 | 0.42 | 0.29 | 0.773 | 1.13 (0.49 ~ 2.60) |
| Liver |  |  |  |  |  |
| No |  |  |  |  | Reference |
| Yes | 1.00 | 0.36 | 2.82 | 0.005 | 2.72 (1.36 ~ 5.45) |
| Bone |  |  |  |  |  |
| No |  |  |  |  | Reference |
| Yes | -0.66 | 0.52 | -1.26 | 0.209 | 0.52 (0.19 ~ 1.44) |
| Her2 expression |  |  |  |  |  |
| 0+ |  |  |  |  | Reference |
| 1+ | -0.48 | 0.45 | -1.07 | 0.285 | 0.62 (0.26 ~ 1.49) |
| 2+ | -0.07 | 0.51 | -0.13 | 0.896 | 0.94 (0.35 ~ 2.52) |
| 3+ | -1.80 | 1.20 | -1.50 | 0.133 | 0.17 (0.02 ~ 1.73) |
| Previous treatment |  |  |  |  |  |
| No |  |  |  |  | Reference |
| Chemotherapy/radiotherapy | 0.43 | 0.39 | 1.10 | 0.270 | 1.54 (0.71 ~ 3.33) |
| Surgery |  |  |  |  |  |
| Radical surgery |  |  |  |  | Reference |
| Partial resection surgery | 0.00 | 0.55 | 0.00 | 0.999 | 1.00 (0.34 ~ 2.97) |
| Diagnostic surgery | -0.33 | 0.59 | -0.56 | 0.576 | 0.72 (0.22 ~ 2.29) |
| Tumor biopsy | 0.91 | 0.57 | 1.60 | 0.110 | 2.48 (0.81 ~ 7.53) |
| Response |  |  |  |  |  |
| CR |  |  |  |  | Reference |
| PR | 0.58 | 0.66 | 0.87 | 0.382 | 1.79 (0.49 ~ 6.56) |
| SD | 1.38 | 0.75 | 1.85 | 0.064 | 3.98 (0.92 ~ 17.15) |
| PD | 1.45 | 0.70 | 2.07 | 0.038 | 4.26 (1.08 ~ 16.79) |
| HR: Hazard Ratio, CI: Confidence Interval | | | | | |

**Table S5 PFS-based multifactorial Cox regression analysis**

| Variables | β | S.E | Z | *P* | HR (95%CI) |
| --- | --- | --- | --- | --- | --- |
|  |  |  |  |  |  |
| Age |  |  |  |  |  |
| ≤60 |  |  |  |  | Reference |
| >60 | -0.18 | 0.33 | -0.53 | 0.593 | 0.84 (0.44 ~ 1.60) |
| Gender |  |  |  |  |  |
| Male |  |  |  |  | Reference |
| Female | 0.16 | 0.33 | 0.48 | 0.629 | 1.17 (0.62 ~ 2.23) |
| Primary lesion |  |  |  |  |  |
| Bladder |  |  |  |  | Reference |
| Ureter/renal pelvis | 0.20 | 0.32 | 0.61 | 0.541 | 1.22 (0.65 ~ 2.29) |
| Histological differentiation |  |  |  |  |  |
| Urothelial carcinoma |  |  |  |  | Reference |
| Urothelial carcinoma with squamous differentiation | 0.03 | 0.40 | 0.07 | 0.943 | 1.03 (0.47 ~ 2.26) |
| Urothelial carcinoma with glandular differentiation | 1.06 | 0.41 | 2.59 | 0.010 | 2.88 (1.29 ~ 6.42) |
| Lymphovascular invasion |  |  |  |  |  |
| No |  |  |  |  | Reference |
| Yes | 1.02 | 0.33 | 3.06 | 0.002 | 2.77 (1.44 ~ 5.33) |
| Tumor size |  |  |  |  |  |
| ≤3cm |  |  |  |  | Reference |
| >3cm | 0.50 | 0.34 | 1.47 | 0.141 | 1.65 (0.85 ~ 3.24) |
| Hypertension |  |  |  |  |  |
| No |  |  |  |  | Reference |
| Yes | 0.17 | 0.35 | 0.49 | 0.628 | 1.19 (0.60 ~ 2.36) |
| Diabetes |  |  |  |  |  |
| No |  |  |  |  | Reference |
| Yes | 0.34 | 0.34 | 1.01 | 0.314 | 1.41 (0.72 ~ 2.74) |
| GFR |  |  |  |  |  |
| ≥60 |  |  |  |  | Reference |
| <60 | -0.11 | 0.32 | -0.33 | 0.740 | 0.90 (0.48 ~ 1.69) |
| Hyperlipidemia |  |  |  |  |  |
| No |  |  |  |  | Reference |
| Yes | -0.35 | 0.46 | -0.77 | 0.442 | 0.70 (0.29 ~ 1.73) |
| Drug type |  |  |  |  |  |
| RC48 + toripalimab |  |  |  |  | Reference |
| RC48 + tislelizumab | 0.63 | 0.31 | 2.02 | 0.043 | 1.88 (1.02 ~ 3.45) |
| Lymph node |  |  |  |  |  |
| No |  |  |  |  | Reference |
| Yes | 1.36 | 0.39 | 3.51 | <0.001 | 3.90 (1.82 ~ 8.34) |
| Lung |  |  |  |  |  |
| No |  |  |  |  | Reference |
| Yes | 0.09 | 0.35 | 0.24 | 0.810 | 1.09 (0.54 ~ 2.18) |
| Liver |  |  |  |  |  |
| No |  |  |  |  | Reference |
| Yes | 0.07 | 0.35 | 0.20 | 0.839 | 1.07 (0.54 ~ 2.12) |
| Bone |  |  |  |  |  |
| No |  |  |  |  | Reference |
| Yes | -0.17 | 0.39 | -0.44 | 0.660 | 0.84 (0.39 ~ 1.81) |
| Her2 expression |  |  |  |  |  |
| 0+ |  |  |  |  | Reference |
| 1+ | -0.21 | 0.35 | -0.58 | 0.560 | 0.81 (0.41 ~ 1.63) |
| 2+ | -0.39 | 0.38 | -1.04 | 0.299 | 0.68 (0.32 ~ 1.41) |
| 3+ | -1.56 | 0.66 | -2.36 | 0.018 | 0.21 (0.06 ~ 0.77) |
| Previous treatment |  |  |  |  |  |
| No |  |  |  |  | Reference |
| Chemotherapy/radiotherapy | 0.53 | 0.32 | 1.62 | 0.106 | 1.69 (0.89 ~ 3.20) |
| Surgery |  |  |  |  |  |
| Radical surgery |  |  |  |  | Reference |
| Partial resection surgery | 0.37 | 0.42 | 0.88 | 0.377 | 1.44 (0.64 ~ 3.27) |
| Diagnostic surgery | -0.05 | 0.41 | -0.12 | 0.902 | 0.95 (0.43 ~ 2.11) |
| Tumor biopsy | 0.27 | 0.45 | 0.60 | 0.549 | 1.31 (0.54 ~ 3.19) |
| Response |  |  |  |  |  |
| CR |  |  |  |  | Reference |
| PR | 0.98 | 0.56 | 1.74 | 0.081 | 2.67 (0.89 ~ 8.02) |
| SD | 1.43 | 0.61 | 2.33 | 0.020 | 4.17 (1.25 ~ 13.92) |
| PD | 1.30 | 0.64 | 2.05 | 0.040 | 3.68 (1.06 ~ 12.80) |
| HR: Hazard Ratio, CI: Confidence Interval | | | | | |
